# Assessing Social Media Data as a Resource for Firearm Research: Analysis of Tweets Pertaining to Firearm Deaths

**DOI:** 10.2196/38319

**Published:** 2022-08-25

**Authors:** Lisa Singh, Carole Roan Gresenz, Yanchen Wang, Sonya Hu

**Affiliations:** 1 Department of Computer Science Massive Data Institute Georgetown University Washington, DC United States; 2 McCourt School of Public Policy School of Health Georgetown University Washington, DC United States; 3 Department of Computer Science Georgetown University Washington, DC United States

**Keywords:** firearms, fatalities, Twitter, firearm research, social media data

## Abstract

**Background:**

Historic constraints on research dollars and reliable information have limited firearm research. At the same time, interest in the power and potential of social media analytics, particularly in health contexts, has surged.

**Objective:**

The aim of this study is to contribute toward the goal of establishing a foundation for how social media data may best be used, alone or in conjunction with other data resources, to improve the information base for firearm research.

**Methods:**

We examined the value of social media data for estimating a firearm outcome for which robust benchmark data exist—specifically, firearm mortality, which is captured in the National Vital Statistics System (NVSS). We hand curated tweet data from the Twitter application programming interface spanning January 1, 2017, to December 31, 2018. We developed machine learning classifiers to identify tweets that pertain to firearm deaths and develop estimates of the volume of Twitter firearm discussion by month. We compared within-state variation over time in the volume of tweets pertaining to firearm deaths with within-state trends in NVSS-based estimates of firearm fatalities using Pearson linear correlations.

**Results:**

The correlation between the monthly number of firearm fatalities measured by the NVSS and the monthly volume of tweets pertaining to firearm deaths was weak (median 0.081) and highly dispersed across states (range –0.31 to 0.535). The median correlation between month-to-month changes in firearm fatalities in the NVSS and firearm deaths discussed in tweets was moderate (median 0.30) and exhibited less dispersion among states (range –0.06 to 0.69).

**Conclusions:**

Our findings suggest that Twitter data may hold value for tracking dynamics in firearm-related outcomes, particularly for relatively populous cities that are identifiable through location mentions in tweet content. The data are likely to be particularly valuable for understanding firearm outcomes not currently measured, not measured well, or not measurable through other available means. This research provides an important building block for future work that continues to develop the usefulness of social media data for firearm research.

## Introduction

### Motivation

Firearm violence is a major and costly public health burden in the United States [1-3], and constraints on the availability of research dollars and reliable information to support firearm research have imposed limits on the ability to gather scientific evidence on effective gun policy [4-7]. At the same time, interest in the power and potential of social media analytics in public health contexts has surged. Several aspects of social media data have heightened their promise as a resource, including the fact that the data are inexpensive to obtain compared with survey data; provide access to continuous, automated, and near–real-time monitoring; and are passively collected in a naturalistic setting as part of an individual’s day-to-day life, eliminating biases inherent to sampling procedures, questionnaires, and recall [8-13]. Such data are, of course, not without their own methodological challenges and limitations, and practices for their ethical and meaningful use are evolving [14-16].

To date, such data have been deployed in firearm-related research in several ways, including to record narratives, sentiment, and emotion around shooting events [17-20]; characterize gun advertisements on social media [21]; and reflect opinions on gun policies and gun control [22,23]. In this paper, we take up the question of how social media data may contribute to understanding firearm-related outcomes. We identify methodological approaches, challenges, and limitations associated with using social media data for understanding a specific firearm outcome—firearm mortality—for which a benchmark measure for comparison is available from a traditional data source. The analysis of firearm mortality is intended to serve as a test of the potential utility of social media data for understanding firearm outcomes not currently measured, not measured well, or not measurable through other available means.

### Assessing the Usefulness of Twitter Data

Specifically, we assessed the usefulness of Twitter data for understanding firearm mortality. Twitter is an online microblogging platform that has >206 million daily active users worldwide and >77 million daily active users in the United States [24]. A key feature of Twitter is its short format: members can only post messages, known as *tweets*, of up to 280 characters. We developed machine learning (ML) classifiers for identifying tweets that pertain to firearm fatalities and compared measures of firearm-fatality discussion volume to firearm-fatality estimates by state from the National Vital Statistics System (NVSS). The NVSS represents one of the few sources of US health-related data with consistently collected and reliable information on a specific gun outcome measured by geographic area. Our goal was to begin to establish a foundation for how social media data may be used by itself or in conjunction with other data resources, such as through data-blending techniques, to improve the information base on which firearm research relies.

## Methods

### Ethics Approval

The institutional review board of Georgetown University reviewed our submission, STUDY00002288, and determined the study to be exempt.

### Overview

Our overarching approach was to compare—within state over time (by month)—measures of firearm-fatality tweet discussion volume with NVSS estimates of firearm fatalities using Pearson linear correlations. Methodologically, with respect to Twitter data, we used a multistage process as described in detail in the following subsections. We first describe our benchmark data and then describe in detail our approach to analyzing Twitter data.

### Benchmark Data

Our benchmark data are NVSS estimates of overall firearm fatalities by state and month for 2017 and 2018. Diagnostic (International Classification of Diseases, Tenth Revision) codes in the NVSS identify mortality from accidental firearm discharges, assaults (homicides) by discharge of firearms, and intentional self-harm (suicides) by firearms. Data are collected nationwide using standardized forms and a set of common procedures to ensure comparability of data across locations.

### Twitter Data

**Figure 1:**
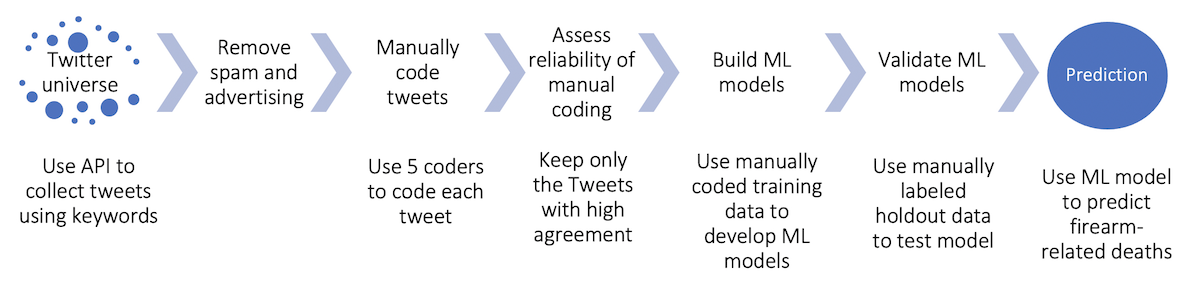
Construction of the Twitter-based gun-related analytic platform. API: application programming interface; ML: machine learning.

We developed a Twitter-based gun-related analytic platform based on content culled from the Twitter Enterprise application programming interface (API) for the 2017-2018 time period through the multistage process depicted in Figure 1. The process consists of 4 stages to prepare the data for ML and 3 stages associated with ML analysis.

The API allows permitted users to access publicly available Twitter content—including tweets; tweet IDs (a unique identification number generated for each tweet); and Twitter profile information such as display name, username, user bio, and publicly stated location—under a developer agreement. The developer agreement requires that the data are used in ways consistent with people’s reasonable expectation of privacy and are not used for developing, creating, or offering commercial services in ways that violate Twitter’s policies. To identify relevant tweets, we hand curated a selected set of keywords and hashtags relating to firearms by looking at a random sample of actual tweets and using keywords identified in previous literature. The query we used to collect data from the API included >200 keywords and hashtags (Multimedia Appendix 1). The data retrospectively collected through the API adds a language label to each tweet. In this study, we used tweets labeled as being written in English.

The initial database we derived from the Twitter API using our curated set of firearm-related keywords and hashtags included >2.3 million tweets for 2017 and 2018. More specifically, we obtained 651,466 tweets from 2017 and 1,675,083 tweets from 2018 (with the increase in the number of tweets over time reflecting larger trends in Twitter discussion on the topic). Given that billions of tweets are posted each year in English on Twitter, the discussion of firearms constitutes a relatively modest size.

Next, in *stage 2*, because social media data are subject to the influence of robots, advertisers, and marketers, the data must be classified and filtered to exclude irrelevant data. We used a multistage process to identify and remove spam (advertising, dead links, pornography, etc). We began by detecting spam using a content-based algorithm because spam can be generated by both humans and bots. The content-based algorithm first looks for website URLs related to known advertising, phishing scheme, malware, gambling, and pornography sites. Our spam blacklist contains >2 million website URLs. The second part of the spam classifier looks for content that maps to standard spam content or differs significantly from other content on the tweet stream being collected [25].

In *stage 3*, we randomly sampled tweets from the resulting data for manual labeling—a process of assigning each tweet a set of characteristics, or features, relevant to the study question. We labeled three firearm-related features of tweets: whether the tweet pertains to (1) firearms (2) fatality or fatalities, or (3) a mass shooting. Our analyses focused on firearm fatality (a combination of characteristics 1 and 2) and mass shooting. We also labeled tweets as an advertisement or irrelevant, spam, or noise and used these labels to further improve our spam classifier and remove identified spam tweets from further analysis.

The manual labeling process relied on crowdsourced, distributed labor through Amazon Mechanical Turk (MTurk) [26-28]. We recognize that varying and evolving views exist regarding the use of this platform [29-31] and were attentive to these considerations in our study design, which was vetted and approved by our institutional review board. We applied best practices, creating as clear and streamlined a task as possible and training MTurk coders through a written instruction guide and with labeling examples (Multimedia Appendix 2) [32]. Recent research confirms that MTurk can be a useful resource for quickly gathering reliable data labels for training ML models when best practices are used [33].

We required each tweet to be labeled by 5 different coders, and we calculated the interrater reliability of labeling across coders. The manual labeling process continued until we reached a threshold number (minimum of 400) of tweets that were labeled as positively identifying a particular characteristic. We found that at least 400 tweets for each class in our ML model was reasonable for building a reliable classifier for our learning tasks. The total number of tweets labeled for each characteristic varied because coders may label one or more characteristics for each tweet, rather than all characteristics for each tweet.

As a means of assessing the manual labeling process, we calculated 2 scores for the set of tweets labeled for each characteristic. The first measured task agreement. For each tweet, we assigned the value of the characteristic being measured according to the majority vote (eg, if, of 5 labelers, 3 chose *yes* for firearm-related and 2 chose *no*, we assigned the value of *yes*) and then calculated the percentage of coders who agreed on this value (in this case, 3/5 = 60%). The task agreement is the average across all tweets for a given characteristic of this score. Second, we calculated a worker performance score for each coder in which the denominator was the total number of characteristics a coder labeled, and the numerator was the number of characteristics labeled for which the coder’s assigned label aligned with the majority vote. We then calculated the average worker performance score for the set of coders who labeled the set of tweets used for measuring each of the characteristics.

Table 1 summarizes the number of tweets that were manually labeled along with task agreement and worker performance score metrics.

As shown in Table 1, we found high rates of task agreement and worker performance for identifying firearm fatalities (97.14% and 97.19% for task agreement and worker performance, respectively) and mass shooting events (95.42% and 94.96% for task agreement and worker performance, respectively). We noted that 50 tweets that were labeled as being firearm-related were not labeled with a mass shooting characteristic. This occurred in our initial experiment of the labeling task. In this experiment, we labeled tweets as being about a mass shooting, homicide, or suicide. If a tweet was labeled as being about a homicide or suicide, we did not ask the labeler to determine whether the tweet was about a mass shooting. In subsequent experiments, we only focused on capturing firearm-related deaths more broadly and mass shootings explicitly to allow for count adjustments. Therefore, for subsequent experiments (we collected a few hundred labels at a time), we always asked labelers to determine whether a tweet about firearm-related fatality was discussing a mass shooting event.

In *stage 4*, we defined *reliably labeled* tweets as those for which there was manual labeling agreement among ≥3 coders. We dropped tweets that had a reliable label of uncertain or were not reliably labeled from further analysis. This means that our training data did not include ambiguous tweets and, therefore, may undercount our characteristics.

The next three stages (*stages 5*, *6*, and *7*) of the process involved firearm-related ML. In *stage 5*, we divided the subset of reliably labeled tweets into training data—on which we built ML classifiers—and holdout data, which were used to validate the classifiers. We randomly selected 80% of reliably labeled tweets for the training data and 20% for the holdout. When building the ML classifiers, we used 5-fold cross-validation on the training data to measure the reliability of the classifiers. Cross-validation is a resampling procedure that allows researchers to determine whether their ML models are generalizable [34-36]. In 5-fold cross-validation, the data set is partitioned into 5 equal subparts (or *folds*). Of the 5 folds, 4 (80% of the data) are used for training, and 1 (20% of the data) is used for testing. This is repeated 5 times so that each fold is part of the training set 4 times and part of the testing set 1 time, and the final accuracy of the model is determined by taking the mean accuracy of all the created models on the testing set.

We began building ML classifiers to identify tweets pertaining to a firearm fatality and to a mass shooting. We minimally preprocessed the data: lowercased text, removed punctuation and URLs, and removed stopwords. We generated a number of features for the ML classifiers: frequent n-grams, words and phrases, and sentiment. The classifiers we compared were random forest, support vector machine, logistic regression, decision tree, and naïve Bayes. In *stage 6*, we validated the classifiers we developed for firearm fatalities and mass shootings in *stage 5* by further testing them on holdout data. We calculated the sensitivity and specificity of the ML model predictions against those of the manually coded firearm-fatality label.

Table 2 summarizes the best-performing ML classifier for each classification task along with the training and holdout data set sizes and a measure of reliability based on the testing data, using our cross-validation approach, and the holdout data. The *F*_1_-score is a weighted average of sensitivity and specificity (precision and recall) that considers both false positives and false negatives. For firearm-related fatality, we had 6045 labeled tweets. For mass shooting, we had 5842 labeled tweets. Because of heavy skews (imbalance) in the training data, we randomly undersampled from the labeled data of the majority label to balance the training and holdout data sets. Table 2 shows the training and holdout data set sizes after this procedure.

We selected random forest classifiers for both firearm fatalities and mass shooting characteristics. The *F*_1_-scores, as shown in Table 2, are high and comparable for the testing and holdout data, indicating a clear ability of the classifiers to generalize beyond the training data set.

*Stage 7* completed the development of our Twitter-based gun-related analytic platform with the third and final piece of the ML analysis. In *stage 7*, we applied the validated classifier to identify firearm-fatality tweets.

**Table 1:** Manually labeled tweet characteristics.

Tweet label	Firearm-related fatality	Mass shooting
Total number of tweets labeled (yes, no, unsure)	5868 (5528, 330, 10)	5478 (419, 5056, 3)
Task agreement, %	97.14	95.42
Worker performance score, %	97.19	94.96

**Table 2:** Machine learning (ML) classifier type and reliability.

Prediction task	Firearm-related fatality	Mass shooting
Training data size, n	1142	1038
Holdout data size, n	286	256
Best ML classifier	Random forest	Random forest
*F*_1_-score: cross-validation, test data, mean (SD)	0.91 (0.017)	0.88 (0.012)
*F*_1_-score: holdout data	0.90	0.88

### Geographic Area Estimation of Twitter Firearm-Fatality Discussion Volume

The NVSS classifies fatalities according to the geographic jurisdiction in which the fatality occurred. Thus, for comparison with the state-level NVSS estimates, the *location of the fatality* being discussed on Twitter is the location of interest (vs the location of the individual who is tweeting). We relied on the tweet content to identify the location of the fatality because location information from either profile information or tweet geocoding (which some users permit) identifies the location of the user (as opposed to location of the fatality).

Importantly, location mentions in tweets primarily refer to city names. In some cases, state name is also mentioned, whereas in other cases, state can be inferred from the city name. To obtain a reasonably sufficient number of tweets per location for estimating area-level fatality discussion volume, we focused on identifying the larger cities mentioned in tweets. Specifically, we identified tweets in our sample that mentioned any of the 250 most populous cities in 2018 (based on US Census data [35]). A limitation of this approach is that it focuses on fatalities in urban areas rather than in rural areas.

We augmented the list of 250 city names with alternative city names commonly used on social media, such as *nyc*, and with city names that contain no spaces between multiple words, such as *sanfrancisco.* We standardized posts—converting the text to lowercase and removing URLs, user mentions (words prefixed with @), and common phrases that may look as though they are city mentions when they are not. An example of a common phrase we removed is *drag queens* because it may be accidently mapped to Queens, New York City, New York. After standardization, we searched the text for city names that matched our location ontology. The majority of city names among the 250 are associated with, and can thus be reliably mapped to, a single state. For our specific set of tweets, there were no cities mentioned that mapped to multiple states.

We summed tweet discussion volume across the most populous cities within a state to create a state-level measure. We constructed state-level estimates for Arizona, California, Colorado, Florida, Georgia, Illinois, Indiana, Kentucky, Louisiana, Maryland, Massachusetts, Michigan, Missouri, Nevada, New Jersey, New York, North Carolina, Ohio, Pennsylvania, Tennessee, Texas, Virginia, Washington, and Wisconsin. We excluded from further analysis those states for which the sample size of tweets was <200 tweets after the mass shooting adjustments (described in the next paragraph) because they are home to only one or only a few of the more populous cities (eg, Idaho, Iowa, Nebraska, and Oregon), and the populous cities in the state are relatively small (eg, Kansas, Alabama, and Arkansas).

The resulting data set, after applying the best ML classifier to identify firearm-fatality tweets and identifying the state of the fatality using location mentions, included 31,747 tweets from 2017 and 44,779 from 2018. We summarized firearm-fatality discussion volume for each state using these data. We then adjusted the state-level estimates of firearm-fatality discussion volume in 3 ways. First, mass shooting events tend to generate disproportionately high levels of discussion, that is, levels of discussion that are far higher than for other less high-profile fatalities. We accounted for the potential distorting influence of mass shooting events on the relationship between a gun fatality and tweet discussion volume by excluding tweets from the location of mass shooting events for a period of 1 week after the event. We based the 1-week exclusion period on observed trends in mass shooting discussion volume. We identified mass shooting events during the time frame of our data using information from the Gun Violence Archive [37], Everytown Research [38], and The Violence Project [39]. Finally, we adjusted our estimates of state-level discussion volume by the percentage of the state-level population that uses Twitter [40].

We tested for serial correlation and found that the NVSS data contained 10 states in our final data set with some moderate serial correlation, and the Twitter data contained 5 states with moderate serial correlation. For this reason, we made each time series stationary by differencing monthly estimates [41]; we refer to this as the *Change* result. For the level correlation, we removed states in which both time series had higher levels of serial correlation because the correlation is valid if one of the time series exhibits serial correlation and the other does not [42]. This issue arose with four states: Georgia, Indiana, Michigan, and North Carolina.

## Results

### Correlation Analysis

Table 3 shows results from our correlation analysis. We estimated the correlation within state by month between the *level* of firearm-fatality discussion volume and the *level* of NVSS-reported fatalities, as well as the correlation within each state in the monthly *change* in discussion volume versus the monthly *change* in the NVSS fatality rate.

**Table 3:** Results of correlation analysisa.

	Discussion volume	Discussion volume adjusted for average state-level Twitter use
LEVEL: Correlation, range	–0.293 to 0.535	–0.289 to 0.537
LEVEL: Correlation, mean; median	0.085; 0.091	0.087; 0.093
CHANGE: Correlation, range	–0.057 to 0.682	–0.059 to 0.688
CHANGE: Correlation, mean; median	0.313; 0.303	0.312; 0.301

^a^Pearson linear correlations are reported.

The correlation between the monthly level of firearm-fatality tweets and the monthly number of fatalities measured by the NVSS is weak (median 0.081) and widely dispersed across states (range –0.31 to 0.54). The correlation between month-to-month changes in firearm fatalities discussed in tweets versus those estimated in the NVSS is moderate (median 0.30) and exhibited less dispersion among states than the monthly level correlations (range –0.057 to 0.68). For the correlation among month-to-month changes in firearm fatalities, almost half (11/24, 46%) of the states have correlations ranging from 0.1 to 0.4. More than a quarter (7/24, 29%) of the states have correlations below this range, and a quarter (6/24, 25%) have correlations above this range. The results for the adjusted discussion volume (second row of Table 3, discussion volume adjusted for Twitter use in the state) are very similar to the unadjusted results, with negligible differences observed in estimated correlation rates.

**Figure 2:**
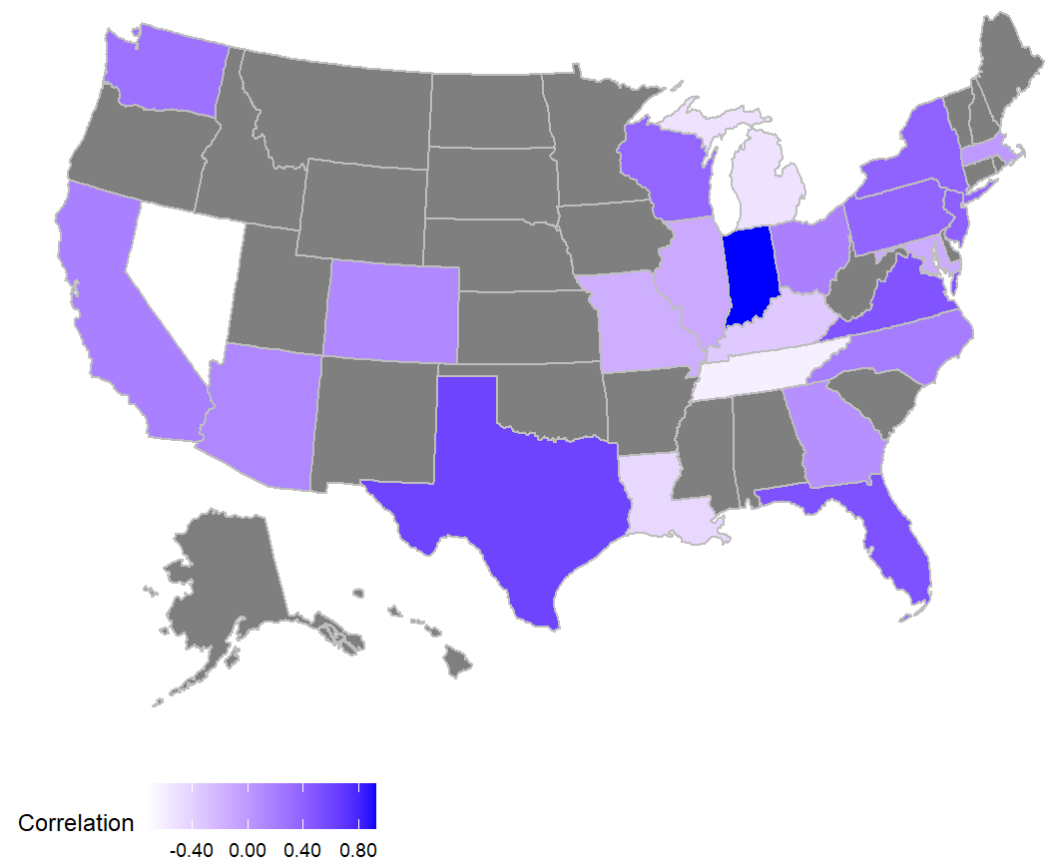
Correlation by state between change in firearm–fatality tweets and change in National Vital Statistics System–estimated firearm fatalities in 2017.

Figure 2 provides additional details for the correlation in monthly changes in fatality discussion volume and NVSS-estimated fatalities, with a depiction of state-by-state (adjusted) correlation rates for 2017. White-shaded states have no correlation. The darker the purple shade of a state, the higher the correlation. The gray-shaded states are those for which we were not able to estimate a Twitter fatality discussion rate (refer to the Twitter Data subsection under Methods). Not unexpectedly, the strength of the correlation seems to be related to the percentage of the state’s population living in one of the most populous cities that we use in our location ontology; for example, one-third or more of the state population in Texas, New York, and Arizona reside in one of the top 100 most populated cities in the state (34%, 44%, and 51%, respectively). These states exhibit some of the highest correlation rates between monthly fluctuation in firearm-fatality discussion volume and NVSS-based fatality estimates. Likewise, Georgia, Michigan, and Maryland are among the states with both the lowest percentage of their population living in more populous cities (5%, 7% and 10%, respectively) and have some of the lowest rates of correlation among the states studied.

By contrast, 47% of Nevada residents live in one of the most populous cities, but the correlation rate in Nevada falls into a lower tier than the correlation rates in Texas, New York, and Arizona. Although we adjusted for mass shooting discussion volume by removing tweets from the week after such an event, the lower correlation observed in Nevada suggests that the adjustment may have been insufficient for capturing the extent of discussion volume distortion in the wake of the mass shooting event in Las Vegas, given the magnitude of the event. Analyzing the data in more detail shows that discussion of this shooting returns at anniversaries (1 year) and when other larger mass shootings occur in other parts of the country, identifying a need for a more extensive adjustment for historically large mass shootings.

### Comparison of Correlations

Furthermore, a comparison of correlations for each state in 2017 versus 2018 shows that states with the largest cities tend to have the most stable correlations; for example, Texas, New York, California, Florida, and Ohio; whereas states with fewer large cities and fewer tweets tend to have higher variation in their correlation estimates; for example, Missouri, Tennessee, South Carolina, and Maryland. An additional factor that is likely to affect the correlation rate is the location within the state of firearm fatalities. To the extent that fatalities within a state are more concentrated in the most populated cities, the correlation between NVSS-estimated fatalities and Twitter discussion volume is expected to be higher.

## Discussion

### Principal Findings

Among the subset of states studied, we found weak-to-moderate correlation between our measure of the level of firearm-fatality tweets and the NVSS-based estimates of the level of firearm fatalities and higher moderate correlation in measures of the month-to-month changes in firearm-fatality tweets and estimated fatalities. As our ontology for Twitter location mentions relies on identification of the 250 most populous cities, our correlation is higher in states in which more of the state’s population was living in one of these cities. We further expected the correlation to be higher in areas where firearm fatalities were concentrated in the most populated cities and found suggestive evidence regarding this point.

A key limitation of this analysis is that we relied on tweets from more populated cities to develop a state-level estimate of discussion volume. Our approach reflected, dually, the limited availability of firearm-fatality data at the city level and the limited availability of location identifiers for tweets. An important feature of this analysis was the need to identify the location of the event being discussed versus the location of the user. In the case of the latter, geocoding of the user profile is advantageous and can provide a state-level identifier, but the former relies only on location mentions within the tweet.

Even with these limitations, the correlation capturing fluctuation in firearm mortality is moderate. We view this as a promising signal for the potential of social media data to provide meaningful information on gun-related outcomes in the future. More specifically, our findings suggest that Twitter data may hold particular value for tracking dynamics in gun-related outcomes. In addition, for location-specific firearm-related outcomes, the data are most valuable for understanding dynamics in relatively populous cities that are identifiable through location mentions in tweet content. Finally, the data are likely to be particularly valuable for understanding firearm outcomes not currently measured, not measured well, or not measurable through other available means. A key advantage of Twitter data is the continuous, automated, and near–real-time monitoring they provide [13]. Once big data infrastructure has been invested in, the data can be relatively easily processed. The initial cost of big data infrastructure can be high if researchers want to stream data for large periods of time. However, for a single study, researchers who can access a server should be able to conduct the analysis at a low cost. Because of this potentially higher investment, we have developed a text analytic portal that allows researchers to construct variables from our social media data [43], thereby enabling future research with these data without the cost of setting up big data infrastructure.

We recognize the need for additional analyses to continue to adapt and extend upon the approach developed and applied in this research, including, for example, work that assesses the reliability of associations over longer time periods. We also note that, unlike survey data that are sampled to be representative of the underlying population, social media data emanate from those who use a particular platform. Although the use of Twitter in the United States is significant (in 2021, nearly a quarter of adults reported using Twitter, and among those who reported using the platform, nearly half said that they use it once a day or more) [44], it is nonetheless also true that rates of social media use are correlated with age and to some extent with other demographic characteristics [44]. Much of the existing analytic work with social media data does not directly deal with this issue. In our approach, we adjusted our estimates for the percentage of Twitter users in each state. Additional statistical adjustments that more completely account for engagement with the platform are important for future work. Furthermore, social media data include limited sociodemographic information about users. Additional methodological strides toward developing robust methods for demographic imputations represent an important dimension of future efforts.

### Usefulness of Social Media Data

The Centers for Disease Control and Prevention [45] describes its public health approach to prevention of violence, including firearm violence, as encompassing four steps: defining and monitoring the problem, identifying risk and protective factors, developing and testing prevention strategies, and assuring widespread adoption [46]. For firearm violence, the first step—building a foundation of information for describing the epidemiology of such violence—requires focused resources and development. In addition to recent developments in survey, administrative, and other data, such as the important efforts by news media and other organizations to track gun violence incidents in significant detail and the advent of data scraping from obituaries [37,47,48], social media data are a promising future source. This research provides an important building block for future work that continues to develop the usefulness of social media data, alone or in conjunction with other data resources, to strengthen the information base on which firearm research relies, and, more generally, contributes to the process of integrating emerging big data algorithms and traditional data sources for behavioral understanding, decision support, and evidence-based public policy.

As we build out the power of social media data for informing public health problems such as firearm violence, several important dimensions need to be kept in mind. The role that social media may play in exacerbating gun violence or spreading trauma related to gun violence cannot be ignored. However, these data can also be used to help target and improve our understanding of those who use guns and allow for new approaches to gun violence–prevention interventions [49]. To use these data to improve public health outcomes and our understanding of human beliefs and behaviors, we must spearhead establishing best practices for using social media data in ethical ways [50-52], as well as understanding representativeness, methodological limitations, and algorithmic biases.

## Appendix

Multimedia Appendix 1 Hashtags, keywords, and phrases used to collect data from the Twitter application programming interface.

Multimedia Appendix 2 Instructions provided to Amazon Mechanical Turk coders.

## Abbreviations

API: application programming interface
MDI: Massive Data Institute
ML: machine learning
MTurk: Amazon Mechanical Turk
NVSS: National Vital Statistics System
