# Supplementary material for: Assessing Social Media Data as a Resource for Firearm Research: Analysis of Tweets Pertaining to Firearm Deaths
Source: J Med Internet Res. 2022 Aug 25;24(8):e38319. doi: 10.2196/38319 (PMC9459834; doi:10.2196/38319)
Supplement: Multimedia Appendix 1 [file jmir_v24i8e38319_app1.pdf]

Appendix 1: Hashtags, Keywords and Phrases Used to Collect Data from Twitter API

#2A	#neveralone	Good guy with a gun
#2ADefenders	#NotAGunOwner	got shot
#2Amerndment	#nra	got shot to death
#2AShallNotBeInfringed	#opencarry	gun accident
#2ndAmendment	#outofthedarkness	gun death
#9mm	#packingheat	Gun owner
#ak47	#pain	Gun rally
#AllGunControlIsRacist	#parkland	gun violence
#AntiNRA	#pewpew	hate myself
#ar15	#pistol	i didn't mean to shoot her
#BanAssaultWeaponsNow	#progun	i didn't mean to shoot him
#BanAutomaticWeapons	#reachout	i didn't mean to shoot it
#BanSilencers	#realconvo	i feel empty
#BAWN	#rifles	i just shot myself
#bethe1to	#RKBA	I'm a gun owner
#bluehelp	#SecondAmendment	it was an accident
#ChooseForward	#selfharm	keep and bear arms
#colddeadhands	#selfinjury	kill myself
#comeandtakeit	#shooting	murder
#commonsensegunlaws	#shotgun	murdered
#ConcealedCarry	#smithandwesson	murders
#Confiscate	#suicide	my life is pointless
#death	#suicideawareness	my suicide letter
#DefendTheSecond	#SuicideIsNeverAnOption	my suicide note
#depression	#suicideloss	need to die
#donttreadonme	#suicideprevention	never wake up
#firearms	#suicidio	not worth living
#FloridaShooting	#targetpractice	Open carry
#girlswhoshoot	#unsuicide	Pistol
#glock	#wellregulatedmilitia	pop a cap
#gun	#worldsuicidepreventionday	Pro-Gun
#GunBan	.45 caliber	pump full of lead
#GunControl	2nd Amendment	put an end to this
#GunControlNow	9 millimeter	ready to jump
#GunDebate	9mm	really need to die
#gungram	accident shooting	Right to bear arms
#gunlife	accidental death	Second Amendment
#gunowner	accidental murder	shoot a cap
#GunOwnerBut	accidental shooting	shoot up
#gunowners	accidentally shot	shooting
#gunporn	accidentally shot myself	Shooting champion
#gunrange	AK-47	shot by accident
#GunReformNow	AR-15	shot to death
#gunrights	be dead	Shotgun
#guns	Bear arms	sleep forever

#GunSafety	better off dead	smith & wesson
#gunsdaily	better off without me	stop the pain
#gunsense	bust a cap	suicidal
#gunsofinstagram	can't do this anymore	suicide
#GunsSaveLives	can't go on	suicide attempt
#GunViolence	Come and take it	suicide pact
#gunweek	Concealed weapon permit	suicide plan
#handgun	CWP	take my own life
#help	didn't mean to shoot	Take the guns
#iamagunowner	die alone	Target practice
#killyourself	don't want to be here	tired of living
#malesuicide	don't want to go on	to take my own life
#mentalhealth	end it all	unintentional death
#mentalhealthawarenessday	end my life	unintentional shooting
#mentalhealthmatters	fall asleep and never wake up	unintentionally shot
#mentalillness	Glock	want to die
#momsdemandaction	go to sleep forever	want to die right now
