# Supplementary material for: Assessing Social Media Data as a Resource for Firearm Research: Analysis of Tweets Pertaining to Firearm Deaths
Source: J Med Internet Res. 2022 Aug 25;24(8):e38319. doi: 10.2196/38319 (PMC9459834; doi:10.2196/38319)
Supplement: Multimedia Appendix 2 [file jmir_v24i8e38319_app2.pdf]

Appendix 2: Mechanical Turk Instructions

Instructions:

Review the definitions and examples, then review the text presented in the box, and answer the questions to determine the text's relevance to gun violence.

Please choose "Not Enough Information" if it is not possible to determine whether the question should be answered Yes or No.

IMPORTANT Please label carefully. We will be using the results of this survey to conduct academic research related to gun violence discussion on social media. We thank you in advance for your attention.

Definitions:

The material describes a gun if:

- The object in question is a firearm
- A firearm is explicitly mentioned in the text

The material mentions a fatal incident if:

- It explicitly states one or more persons died as the result of the use of a gun

The material describes a homicide if:

- The victim died as a result of another individual's intentional actions

The material describes a suicide if:

- The victim dies as a result of their own intentional actions to end their life

The material describes a mass shooting if:

- If four or more people are shot (injured or killed) during the shooting incident

The material is considered to be irrelevant, noise or spam if:

- Is NOT gun related
- Is NOT gun death related
- It asks people for their personal information
- It contains harmful or inappropriate content/links including malware, phishing, or pornography
- It is not understandable, e.g., not a complete sentence, not human languages, etc.

The material is considered to be an advertisement if:

- It has the hashtag "#ad"
- It is an advertisement for products or services
- It leads people to click, view, buy, get involved in any commercial goods and services
- It is a sponsored content – the poster of the statement is likely getting paid for sharing product information

<u>Examples</u>:

Gun:

- Precision Built Rifles by Gunmaker Store. Get yours and start customizing today!

Fatal Incident:

- Mother killed in #KansasCity shooting had 7-year-old daughter and college hopes.

Accidental Death:

- Four-year-old John Doe died after finding a loaded gun in a bedroom closet and discharging it.

Not Gun/Firearm, Gun Fatality, or Gun Accidental Death related:

- Getting my pump on in the weightroom #guns
- Using my favorite Call of Duty #gun killing newbs

Examples that are irrelevant, noise, or spam:

- Sometimes I like to be in front of the camera especially in action #shortfilms like Yun-Ho by Hermann Swaiss where i was honored to play and amazing scene
- Free US S&H! #SaturdayMotivation #gymday #fit #gymmotivation #keto #goals #bodybuild #weightlifter #deadlifts #core #energy #legsday #squats #guns #beastmode #whynot #train #twitch #gamer #justdoit #gymgirl #gymrat #mma #fight
- This meme is old, but it still cracks me up every time I see it :rolling_on_the_floor_laughing::joy: #videogames #gamingmeme #yoshi #guns #funny #nintendo

Examples that are ads:

- You can always find great deals at Bullseye Sport Guns & Ammo! Take time to browse through our Wide Range of AR Complete Uppers. Lock and load with us today!
- Showmaster's Gun Show returns to The Convention Center! Buy, sell, & trade! 650 tables of shotguns, rifles, handguns, swords, knives, war paraphernalia - AND MUCH MORE!! Sat, Feb 1, 9am–5pm. Sun, Feb 2, 9am–4pm

<u>Questions</u>:

- Does the text describe a death (fatal incident)? (Yes, No, Not Enough Information)
  - (If yes - describes death) Does the text describe a gun related death? (Yes, No, Not Enough Information)
- Does the text describe a mass shooting? (Yes, No, Not Enough Information)

- Is the text an advertisement? (Yes, No, Not Enough Information)
- Is the text irrelevant, noise, or spam? (Yes, No, Not Enough Information)
